# Transcriptome analysis of pig intestinal cell monolayers infected with *Cryptosporidium parvum* asexual stages

**DOI:** 10.1186/s13071-018-2754-3

**Published:** 2018-03-12

**Authors:** Marzieh Ezzaty Mirhashemi, Farzad Noubary, Susan Chapman-Bonofiglio, Saul Tzipori, Gordon S. Huggins, Giovanni Widmer

**Affiliations:** 1 Clinical and Translational Institute, Sackler School of Biomedical Sciences, Tufts University, 136 Harrison Avenue, Boston, Massachusetts 02111 USA; 2 Cummings School of Veterinary Medicine at Tufts University, Building 20, 200 Westborough Avenue, North Grafton, Massachusetts 01536 USA

**Keywords:** Transcriptome, *Cryptosporidium parvum*, *Sus scrofa*, RNA-Seq

## Abstract

**Background:**

Human cryptosporidiosis is caused primarily by two species of apicomplexan protozoa, *Cryptosporidium parvum* and *C. hominis*. In cultured cell monolayers, the parasite undergoes two generations of asexual multiplication (merogony). However, the proportion of parasites completing the life-cycle is low and insufficient to sustain continuous propagation. Due to the intracellular location of meronts and later life-cycle stages, oocyst and sporozoites are the only forms of the parasite that can readily be isolated.

**Results:**

Research on the replicating forms of *Cryptosporidium* parasites and their interaction with the host cell remains challenging. Based on an RNA-Seq analysis of monolayers of pig epithelial cells infected with *C. parvum*, here we report on the impact of merogony on the host’s gene regulation. Analysis of the transcriptome of infected and uninfected monolayers demonstrates a significant impact of the infection on host cell gene expression. A total of 813 genes were differentially expressed. Functional terms significantly altered in response to infection include phosphoprotein, RNA binding and acetylation. Upregulation of cell cycle pathways indicates an increase in mitosis. Notably absent from differentially enriched functional categories are stress- and apoptosis-related functions. The comparison of the combined host-parasite transcriptome reveals that *C. parvum* gene expression is less diverse than the host cell transcriptome and is highly enriched for genes encoding ribosomal functions, such as ribosomal proteins.

**Conclusions:**

These results indicate that *C. parvum* infection significantly changes host biological functions and provide new insight into gene functions driving early *C. parvum* intracellular development.

**Electronic supplementary material:**

The online version of this article (10.1186/s13071-018-2754-3) contains supplementary material, which is available to authorized users.

## Background

Cryptosporidiosis in humans is caused primarily by *Cryptosporidium parvum* and *C. hominis* (Phylum Apicomplexa)*.* Infection with these protozoans is the second-most frequent cause of diarrhea in infants living in developing nations [1] and is relatively common in immunocompromised individuals [2, 3]. As typically observed with other coccidia, rapid multiplication of the parasite in the intestinal epithelium compromises intestinal function and leads to diarrhea and malabsorption. Although numerous publications have described modifications of the original method for culturing *Cryptosporidium* [4, 5], our ability to grow these parasites in cell monolayers remains unsatisfactory. Our knowledge of the interaction between host cell and parasite is primarily based on the annotation of the *Cryptosporidium* genome, which has revealed the absence of several biosynthetic pathways and inferred the dependence of the replicating parasite on host cell metabolites [6].

Studying the interaction of *Cryptosporidium* parasites with the host cell remains a difficult undertaking. Parasite development is not synchronous, the proportion of infected monolayer cells is variable and difficult to measure. As a consequence, compared to the oocyst stage, intracellular stages have infrequently been studied, particularly later developmental stages. The transcriptional response of cell monolayers to the presence of *C. parvum* meronts has been investigated with microarrays and reverse-transcription (RT) PCR [7–12]. Studies in monolayers of human HCT-8 cells infected with *C. parvum* have uncovered morphological changes reminiscent of apoptosis [13, 14], reported heat-shock and inflammatory response [7], cytoskeleton modifications [15] and modifications of the host cell membrane [16]. RNA-Seq has recently been used to analyze the *C. parvum* transcriptome in cell monolayers and in experimentally infected calves, but to date no analysis of these data appears to have been published. Here, we report on the analysis of the transcriptional response of pig intestinal epithelial cells to the initial stage of *C. parvum* merogony and compare functional properties of the host and parasite transcriptome in the early phase of merogony.

## Methods

### Parasites and cell lines

#### *Cryptosporidium parvum* oocysts

Fecal samples from diarrheic calves raised in Woodstock, Connecticut, were screened for the presence of *Cryptosporidium* oocysts using acid-fast stained fecal smears. One sample with a high concentration of oocysts (3 × 10^7^ oocysts/ml feces) was selected. Oocysts were extracted on a density gradient of 15–30% Nycodenz (Alere Technologies, Oslo, Norway) as described previously [17]. Oocyst concentrations were determined using a hemocytometer at 400× magnification. The species of this isolate was confirmed using BLAST analysis of sequences obtained as described in the following paragraph. Of 10 randomly selected 101-nt RNA-Seq reads obtained from one of the infected monolayers and which mapped to the *C. parvum* IOWA genome, 8 sequences were 100% identical to *C. parvum* sequences in the NCBI nucleotide collection, one sequence was 100% identical to *C. parvum* and to *C. hominis*, and for one sequence no significant *Cryptosporidium* hits were found. Based on this analysis, and consistent with the host origin of the oocysts, we conclude that the isolate used in these experiments is *C. parvum*. The transcriptome of oocysts of isolate TU114 [18] was analyzed using RNA-Seq as described below.

#### Infection of cell monolayers

Monolayers of pig jejunal epithelial cells (IPEC-J2) [19] were grown to near-confluence in four 75 cm^2^ flasks using DMEM/F12 media (Life Technologies) with 5% fetal bovine serum. Oocysts were surface-sterilized with 10% bleach. Monolayers were infected [13, 14] with a dose equivalent to 1.4 × 10^5^ oocysts/cm^2^, which corresponds to approximately 1 oocyst/cell. Cultures were incubated at 37 °C in a humidified incubator with 5% CO_2_ for 24 h.

### Immunofluorescence

Immunofluorescence was used to confirm the infection of the cell monolayers. Infected and control monolayers were washed 3× with PBS to remove unexcysted oocysts. Monolayers were then fixed with methanol at room temperature for 15 min. Following fixation, monolayers were washed 3 times with PBS and blocked with DMEM medium supplemented with 5% fetal bovine serum at room temperature for 15 min. Following one more wash with PBS, 100 μl of 5 μg/ml monoclonal antibody 2E5 (a gift from Dr. Abhineet Sheoran) conjugated with Fluorescein-5-Isothiocyanate (FITC) was added to each monolayer and the plates incubated at room temperature for 30 min. Antibody 2E5 reacts with intracelluar stages of *C. parvum*. Plates were dried in the dark and read with an inverted epifluorescent microscope using a 40× objective.

### Molecular biology methods

Total RNA was extracted from infected and uninfected cells with a PureLink RNA Mini Kit (Life Technologies). Samples were lysed and homogenized in the presence of guanidinium isothiocyanate. After homogenization, ethanol was added to the sample. The sample was then processed through a Spin Cartridge containing a clear silica-based membrane provided in the kit, to which the RNA binds. Impurities were removed by subsequent washing with the wash buffers provided. Purified RNA was eluted in RNAse-free water. DNA was removed using DNase (DNA-free^TM^, Life Technologies). The quality of the RNA was assessed by reading the 260/280 nm absorbance ratio. The RNA Integrity Number (RIN) was determined using an Agilent Bioanalyzer 2100. An Illumina (TruSeq Stranded RNA library) kit was used to make the cDNA libraries from RNA extracted from four infected and four uninfected 75 cm^2^ cell monolayers harvested 24 h post-infection. The eight cDNA libraries were subjected to cluster generation and single-end 100-nucleotide sequencing on an Illumina Hi-Seq 2500 at the Tufts Genomics core facility (tucf.org). Sequencing data were deposited in the European Nucleotide Archive under project accession number PRJEB17685.

### Gene expression analysis

The *S. scrofa* reference genome and annotation (susScr3) was downloaded from iGenome (http://support.illumina.com/sequencing/sequencing_software/igenome.html). The *C. parvum* IOWA isolate [20] genome and annotation (version 34) was downloaded from the *Cryptosporidium* Genomics Resource database CryptoDB.org [21]. Each RNA-Seq sample was randomly subsampled to 7 million reads to obtain a dataset which could more easily be processed with available computational resources. Sequences were converted from FASTQ to FASTA format and subsampled in *mothur* [22]. Reads were mapped to the pig genome using HiSat2 [23] as implemented in Galaxy (usegalaxy.org) [24]. Reads that did not align to the pig genome were subsequently mapped, also with HiSat2, to the *C. parvum* IOWA genome to estimate the proportion of parasite transcripts in relation to the combined host-parasite transcriptome. A table of FPKM (fragments per kilobase of transcript per million mapped reads) for the RNA-Seq data which mapped to the *S. scrofa* genome was created with Cufflinks [25]. Cufflinks returned FPKM values for 4939 *S. scrofa* genes. The correlation between FPKM values from replicate samples was visualized as shown in Additional file 1: Figure S1. Differentially expressed genes were identified using DESeq2 [26] as implemented in Galaxy using one HiSat2 output file for each of the eight transcriptome samples. Principal Coordinates Analysis (PCoA) was performed with GenAlEx [27]. Pairwise distances between samples were calculated using the SSR metric. This distance was calculated by adding the square of the difference in FPKM between two samples over all genes. Alternatively, the Euclidian distance was used. Analysis of Similarity (ANOSIM) [28] was used to test the significance of the clusters revealed by PCoA. ANOSIM was run in *mothur*.

Program LefSe as implemented in Galaxy at huttenhower.sph.harvard.edu/galaxy/ was used for Linear Discriminant Analysis (LDA) to identify marker genes, i.e. genes that best explain the difference between infected and control transcriptome samples. LDA was applied to a table of 8 samples × 4939 *S. scrofa* genes. The 8 × 4939 fields of the table represented FPKM values, where zero indicated that no sequence mapped to a particular gene.

Gene function and enrichment analyses were performed with DAVID [29]. The False Discovery Rate method [30] and Bonferroni correction was used to identify differentially transcribed genes or enriched functions. Shannon diversity is defined as -Σ p_i_ * ln(p_i_), where the sum is over all genes and p_i_ is the proportion of FPKM value of gene i. pi was calculated by dividing each gene’s FPKM by the sum of all FPKM values in a sample, such that Σ p_i_ over all genes is equal to 1. Diversity calculations were performed in Microsoft Excel.

## Results

### Host cell and parasite transcriptome

The number of sequences from four infected and four control monolayers mapping to the *S. scrofa* and *C. parvum* genome is shown in Table 1. RNA-Seq data from *C. parvum* oocysts were also mapped to the two genomes as a quality control. As expected, the proportion of oocyst reads mapping to the *S. scrofa* genome was close to zero (1037/7 × 10^6^ = 0.014%), whereas 83.7% of oocyst reads mapped to the *C. parvum* genome (Table 1). From the mapping statistics, it is possible to estimate the proportion of parasite transcripts in relation to the host transcriptome. According to Table 1, the average number of RNA-Seq reads that aligned uniquely and > 1 time to the *C. parvum* genome is 117,397 (*n* = 4; SD = 7416), which is 2.20% of the number of reads aligning to the *S. scrofa* genomes (5,322,039; *n* = 8; SD = 60,029). The extent of infection of IPEC-J2 cell monolayers at 24 h post-infection was evaluated using immunoflourescence (Additional file 2: Figure S2). The immunofluorescence pattern indicated that about 50% of cells were infected. Based on these data, we estimate that 4.40% (2.20% × 2) of the transcripts in infected cells originate from *C. parvum*. Given that the *S. scrofa* genome counts about 12 times more genes than the *C. parvum* genome (46,161 *vs* 3880 genes), and the number of pig transcripts was 45.3 time higher than *C. parvum* transcripts, the host cell transcript is approximately four times more abundant on a per-gene basis than the parasite transcriptome. Mean Shannon diversity for the host cell transcriptome was 6.328 (SD = 0.0498, *n* = 8). For the parasite transcriptome, diversity was significantly lower (mean = 6.265, SD = 0.0289, *n* = 4; *t* = -2.299, *P* = 0.044).

**Table 1:** Summary of sequence reads mapping to the genome of *Sus scrofa* and *Cryptosporidium parvum*^a^

	*S. scrofa*				*C. parvum* ^b^			
Sample	Overall align rate (%)	Uniquely aligned seqs	Aligned > 1 time	Unaligned seqs	Overall align rate (%)	Uniquely aligned seqs	Aligned > 1 time	Unaligned seqs
1-infec	75.1	4,649,709 (66.4%)	604,112 (8.6%)	1,746,179 (25.0%)	7.2	122,751 (7.0%)	2,988 (0.17%)	1,620,440 (92.8%)
2-infec	75.7	4,688,821 (66.9%)	611,073 (8.7%)	1,700,106 (24.3%)	6.3	104,797 (6.2%)	2,966 (0.17%)	1,592,343 (93.7%)
3-infec	74.9	4,637,493 (66.2%)	608,208 (8.7%)	1,754,299 (25.1%)	6.7	114,070 (6.5%)	3,036 (0.17%)	1,637,193 (93.2%)
4-infec	75.4	4,657,387 (66.5%)	618,759 (8.8%)	1,723,854 (24.6%)	6.9	115,771 (6.7%)	3,210 (0.19%)	1,604,873 (93.1%)
5-ctrl	77.0	4,783,118 (68.3%)	606,596 (8.7%)	1,610,286 (23.0%)	0	39 (0%)	5 (0%)	1,610,242 (100%)
6-ctrl	76.8	4,779,410 (68.2%)	599,281 (8.6%)	1,621,309 (23.2%)	0	50 (0%)	2 (0%)	1,621,257 (100%)
7-ctrl	76.4	4,746,496 (67.8%)	604,476 (8.6%)	1,649,028 (23.6%)	0	24 (0%)	2 (0%)	1,649,002 (100%)
8-ctrl	76.9	4,775,767 (68.2%)	605,605 (8.6%)	1,618,628 (23.1%)	0	30 (0%)	6 (0%)	1,618,592 (100%)
oocysts	–	1037 (0%)	–	–	–	5,859,947 (83.7%)	–	–

^a^Sequences were rarified to 7 × 10^6^/sample

^b^Sequence that did not align to the pig genome were aligned to *C. parvum*

We compared the host cell and parasite transcriptome in relation of function. The results of a function enrichment analysis for the 100 *C. parvum* and 100 *S. scrofa* genes with the highest mean FPKM is shown in Tables 2 and 3, respectively. The results show that the parasite transcriptome encodes primarily functions annotated as ribosome biogenesis and translation. The analogous analysis to identify enriched functions was performed with the 100 *S. scrofa* genes with highest mean FPKM (Table 3). This analysis reveals a similar pattern of enriched ribosomal functions. However, in contrast to the *C. parvum* transcriptome, other enriched functions such as “acetylation”, “Ubl conjugation” and functions related to the extracellular compartment were also found.

**Table 2:** Significantly enriched functions in the *C. parvum* intracellular transcriptome based on the analysis of 100 genes with the highest FPKM values

Category	Term	Gene count	Fold enrich.	Bonferroni^a^	Benjamini^a^	FDR^a^
UP_KEYWORDS	Ribosomal protein	69	33.01	*1.09E-106*	*1.09E-106*	*3.34E-105*
UP_KEYWORDS	Ribonucleoprotein	69	26.80	*1.88E-95*	*9.40E-96*	*5.77E-94*
GOTERM_MF_DIRECT	GO:0003735~structural constituent of ribosome	66	17.70	*8.23E-93*	*8.23E-93*	*3.24E-91*
GOTERM_BP_DIRECT	GO:0006412~translation	64	10.83	*1.66E-75*	*1.66E-75*	*7.35E-74*
KEGG_PATHWAY	cpv03010: Ribosome	69	7.38	*1.67E-72*	*1.67E-72*	*9.46E-71*
GOTERM_CC_DIRECT	GO:0005840~ribosome	57	14.52	*3.65E-69*	*3.65E-69*	*2.50E-67*
INTERPRO	IPR008991: Translation protein SH3-like domain	7	24.82	*7.81E-06*	*7.81E-06*	*5.00E-05*
INTERPRO	IPR011332: Ribosomal protein, zinc-binding domain	6	28.36	*5.23E-05*	*2.61E-05*	*3.35E-04*
INTERPRO	IPR014722: Ribosomal protein L2 domain 2	6	28.36	*5.23E-05*	*2.61E-05*	*3.35E-04*
GOTERM_CC_DIRECT	GO:0015935~small ribosomal subunit	6	12.99	*2.06E-04*	*1.03E-04*	*0.01*
GOTERM_CC_DIRECT	GO:0015934~large ribosomal subunit	5	17.32	*3.73E-04*	*1.24E-04*	*0.03*

^a^Significant values are italicized

**Table 3:** Significantly enriched functions in the host cell transcriptome based on the analysis of 100 *S. scrofa* genes with the highest FPKM values

Category	Term	Gene count	Fold enrich.	Bonferroni^a^	Benjamini^a^	FDR^a^
UP_KEYWORDS	Ribosomal protein	38	79.30634	9.3879E-62	9.3879E-62	*8.3E-61*
UP_KEYWORDS	Ribonucleoprotein	38	65.58024	6.2878E-58	3.1439E-58	*5.56E-57*
KEGG_PATHWAY	ssc03010: Ribosome	41	28.99698	5.0057E-50	5.0057E-50	*6.79E-49*
GOTERM_MF_DIRECT	GO:0003735~structural constituent of ribosome	41	29.07368	4.3664E-48	4.3664E-48	*4.59E-47*
GOTERM_BP_DIRECT	GO:0006412~translation	34	27.72683	2.8412E-37	2.8412E-37	*1.1E-36*
UP_KEYWORDS	Acetylation	37	18.14443	3.5317E-34	1.1772E-34	*3.12E-33*
GOTERM_CC_DIRECT	GO:0022627~cytosolic small ribosomal subunit	18	71.36052	3.98E-26	3.98E-26	*4.28E-25*
GOTERM_CC_DIRECT	GO:0022625~cytosolic large ribosomal subunit	19	53.41227	5.2961E-25	2.648E-25	*5.69E-24*
UP_KEYWORDS	Phosphoprotein	33	9.461551	5.8773E-21	1.4693E-21	*5.2E-20*
GOTERM_CC_DIRECT	GO:0070062~extracellular exosome	43	4.181397	1.2331E-15	4.1104E-16	*1.33E-14*
GOTERM_CC_DIRECT	GO:0005925~focal adhesion	21	13.99527	1.3908E-15	3.477E-16	*1.5E-14*
GOTERM_CC_DIRECT	GO:0005840~ribosome	13	45.68154	3.6801E-15	7.3603E-16	*3.96E-14*
UP_KEYWORDS	Ubl conjugation	17	19.18998	4.3965E-14	8.7708E-15	*3.89E-13*
UP_KEYWORDS	Isopeptide bond	15	24.92816	8.793E-14	1.4655E-14	*7.77E-13*
GOTERM_MF_DIRECT	GO:0044822~poly(A) RNA binding	26	5.315667	3.7013E-10	1.8506E-10	*3.89E-09*
UP_KEYWORDS	Cytoplasm	21	7.332953	4.9698E-10	7.0997E-11	*4.4E-09*
GOTERM_CC_DIRECT	GO:0016020~membrane	24	5.248581	4.9125E-09	8.1875E-10	*5.28E-08*
GOTERM_BP_DIRECT	GO:0002181~cytoplasmic translation	7	40.70384	4.3892E-06	2.1946E-06	*1.7E-05*
GOTERM_CC_DIRECT	GO:0015935~small ribosomal subunit	4	123.6916	0.0002591	3.7018E-05	*0.002786*
GOTERM_BP_DIRECT	GO:0000028~ribosomal small subunit assembly	5	47.76471	0.0009287	0.00030966	*0.003596*
GOTERM_BP_DIRECT	GO:0000027~ribosomal large subunit assembly	5	39.33564	0.00216972	0.00054287	*0.008406*
GOTERM_MF_DIRECT	GO:0003729~mRNA binding	7	13.09892	0.00146297	0.0004879	*0.015379*
GOTERM_MF_DIRECT	GO:0098641~cadherin binding involved in cell-cell adhesion	4	67.36585	0.00221329	0.00055378	*0.023274*
UP_KEYWORDS	Glycoprotein	12	5.067748	0.00274581	0.00034364	*0.024321*
GOTERM_CC_DIRECT	GO:0031012~extracellular matrix	7	12.02557	0.00238484	0.00029842	*0.025667*

^a^Significant values are italicized

**Fig. 1:**
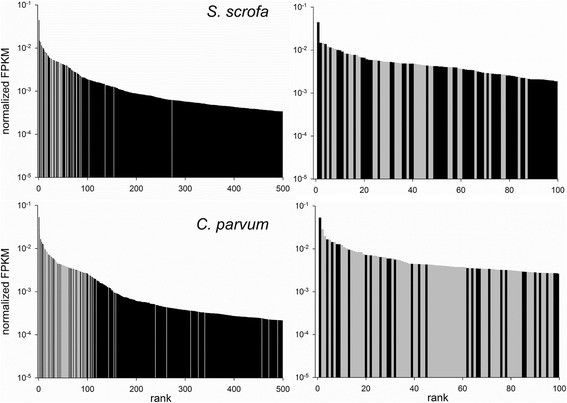
Rank abundance analysis of host and parasite transcriptome. In each graph, host and parasite genes are ranked from left to right in order of diminishing FPKM. Each vertical bar represents a gene. Grey and black bars represent genes encoding ribosomal functions and non-ribosomal functions, respectively. The 500 genes with the highest FPKM are represented in the two bar graphs on the left: top, *S. scrofa*; bottom, *C. parvum*. To convey a clearer view of the function of highly transcribed genes in relation to ribosome-related functions, the 100 genes with the highest FPKM value are shown right. Note the high proportion of genes encoding ribosomal function in *C. parvum* as compared to the host cell transcriptome. The steeper slope of the ranked *C. parvum* FPKM values is consistent with a lower Shannon diversity identified as described in the text

The results of the enrichment analysis of highly expressed genes are consistent with the slightly, but significantly, higher diversity of the host cell transcriptome described above. Whereas in the parasite transcriptome functions relating to ribosome or translation are enriched, in the host other functions were also enriched. The difference in function diversity is represented in Fig. 1. In the bar graphs genes are ranked from left to right in order of diminishing FPKM. Genes encoding ribosome-related functions are represented with light grey. The juxtaposition of the host and parasite transcriptome clearly illustrates the higher proportion of ribosomal functions in highly expressed genes in the parasite transcriptome, as compared to the transcriptome of the host. Moreover, the plots also show the difference in FPKM diversity which is apparent as a more negative slope in the *C. parvum* FPKM rank-abundance plot, as compared to the *S. scrofa* plot.

### Differentially expressed *S. scrofa* genes in infected and control monolayer cells

**Fig. 2:**
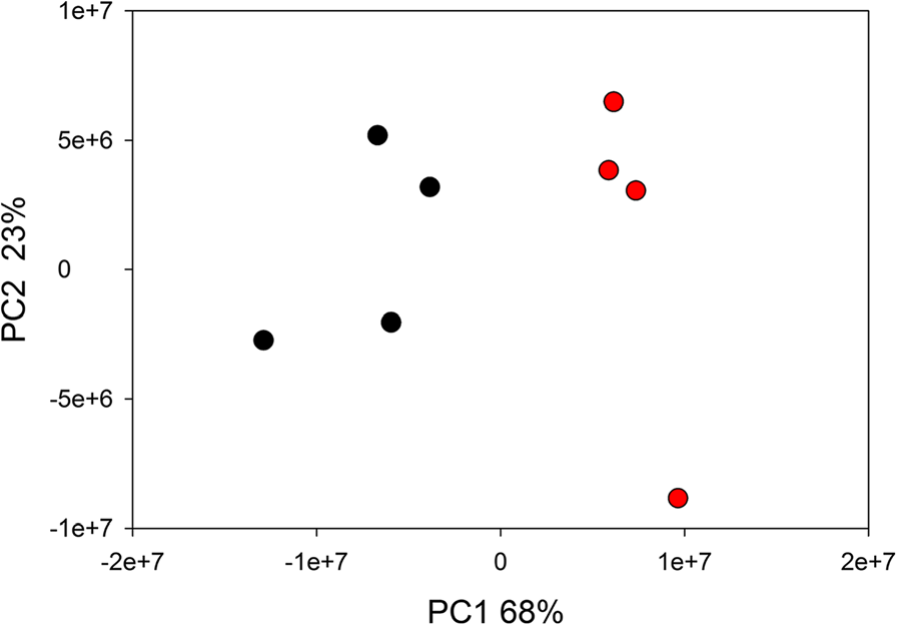
Principal Coordinates Analysis of FPKM data from eight cell monolayers. Analysis is based on FPKM values from 4939 *S. scrofa* genes. Pairwise distances were calculated using the SSR metric. Each black and red symbol represents the transcriptome of an uninfected and an infected monolayer, respectively. Clustering by treatment is statistically significant according to ANOSIM

Having gained insight into the profile of the host cell and parasite transcriptome in infected cells, we investigated whether the host cell transcriptome is affected by the infection with *C. parvum*. PCoA was used to visualize the global difference between the transcriptome of infected and control IPEC-J2 monolayers. PCoAs based on Euclidian distance or on SSR distance [31] gave a similar clustering of infected and control samples. Figure 2 shows the plot based on SSR distance. As apparent from the PCoA, Analysis of Similarity (ANOSIM) confirmed that clustering according to the experimental treatment (infected *vs* control) is significant (*R* = 0.864, *P* = 0.028).

We used DESeq2 to identify *S. scrofa* genes which are significantly up- or downregulated in response to the infection. A total of 810 host genes were found to be differentially expressed at a FDR < 0.05 (Additional file 3: Table S1) in a comparison of four infected and four control monolayers at 24 h post-infection. The result of a function enrichment analysis of genes overexpressed in infected cells found several terms associated with cell division, possibly reflecting damage to the monolayer leading to loss of contact inhibition and resumption of mitosis (Table 4). However, in cells that remained in the monolayer, no transcriptional changes indicative of stress or apoptosis were identified.

**Table 4:** Significantly enriched functions in upregulated *S. scrofa* genes in *C. parvum* infected cell monolayers

Category	Term	Gene count	Fold enrich.	Bonferroni^a^	Benjamini^a^	FDR^a^
UP_KEYWORDS	Phosphoprotein	54	3.67	*7.79E-14*	*7.79E-14*	*4.33E-13*
GOTERM_MF_DIRECT	GO:0044822~poly(A) RNA binding	59	3.24	*4.97E-13*	*4.97E-13*	*1.71E-12*
UP_KEYWORDS	Acetylation	39	4.53	*2.73E-12*	*1.36E-12*	*1.50E-11*
UP_KEYWORDS	Cytoplasm	40	3.31	*2.34E-08*	*7.80E-09*	*1.29E-07*
GOTERM_CC_DIRECT	GO:0070062~extracellular exosome	80	1.93	*1.97E-06*	*1.97E-06*	*8.38E-06*
GOTERM_CC_DIRECT	GO:0005737~cytoplasm	97	1.73	*6.18E-06*	*3.09E-06*	*2.63E-05*
GOTERM_CC_DIRECT	GO:0005925~focal adhesion	24	3.96	*1.23E-05*	*4.10E-06*	*5.23E-05*
GOTERM_CC_DIRECT	GO:0005634~nucleus	94	1.72	*1.74E-05*	*4.36E-06*	*7.41E-05*
UP_KEYWORDS	Nucleus	46	2.35	*3.27E-05*	*8.17E-06*	*1.80E-04*
UP_KEYWORDS	Ubl conjugation	18	4.82	*4.68E-05*	*9.35E-06*	*2.57E-04*
UP_KEYWORDS	Cell cycle	10	8.51	*4.86E-04*	*8.09E-05*	*0.00267*
GOTERM_CC_DIRECT	GO:0005654~nucleoplasm	49	2.04	*8.31E-04*	*1.66E-04*	*0.00353*
UP_KEYWORDS	Isopeptide bond	13	5.12	*0.00198*	*2.83E-04*	*0.01090*
KEGG_PATHWAY	ssc03040: Spliceosome	16	4.00	*0.00189*	*0.00189*	*0.01135*
KEGG_PATHWAY	ssc04110: Cell cycle	15	4.21	*0.00223*	*0.00111*	*0.01335*
GOTERM_MF_DIRECT	GO:0003723~RNA binding	18	3.49	*0.00627*	*0.00314*	*0.02160*
UP_KEYWORDS	Cell division	8	9.46	*0.00398*	*4.98E-04*	*0.02191*
UP_KEYWORDS	Glycoprotein	26	2.60	*0.00575*	*6.41E-04*	*0.03168*
GOTERM_CC_DIRECT	GO:0005794~Golgi apparatus	25	2.62	*0.00957*	*0.00160*	*0.04088*

^a^Significant values are italicized

The overexpression of cell cycle related pathways is consistent with a pathway analysis of the same set of up- and downregulated genes. Spliceosome and Cell cycle were the only two KEGG pathways significantly enriched in the infected samples (FDR < 0.05; Additional file 4: Table S2). These terms do not feature in the analogous pathway analysis of the genes upregulated in uninfected monolayers (Additional file 5: Table S3). Corroborating the analysis of enriched gene function, the function of 39 genes significantly overexpressed according to LEFse analysis [32] broadly overlapped with those shown in Table 4; phosphoprotein was again the highest scoring function with a 7.5-fold enrichment and an FDR value of 0.003. Acetylation, focal adhesion and glycoprotein also featured on the list of ten function terms identified by LEFse.

## Discussion

The analysis of transcriptional changes in cells infected with *C. parvum* is providing new insights into the host response to the infection. Previous studies have shown that apoptosis of host epithelial cells is both induced and inhibited by the infection of *C. parvum* [12, 14, 33–35]. The data presented here support the latter. Understanding the role that apoptosis plays in the pathogenesis of cryptosporidiosis is relevant, because it may help explain the mechanisms leading to diarrhea and blunting of intestinal villi, which are hallmarks of cryptosporidiosis [36]. Since different studies use different cell lines, parasite dose, incubation time and analytical techniques, it is not surprising that they lead to different conclusions. IPEC-J2 cells have been reported to be capable of undergoing apoptosis [37], indicating that deficiency in apoptosis pathways are not at the root of our observations. IPEC-J2 cells originate from a pig, as opposed the more commonly used human cell lines such as HCT-8. In addition, these cells were isolated from the jejunal epithelium, as opposed to the colon, from where HCT-8 and CaCo-2 cells originate. We chose to work with IPEC-J2 cells because of our interest in eventually extending the transcriptome analyses to *C. parvum* development *in vivo* and comparing gene expression *in vivo* and in culture. Germ-free neonatal piglets are highly susceptible to various *Cryptosporidium* species [38, 39]. Moreover, the large size of the GI tract make them ideal models for comparing *Cryptosporidium* development *in vivo* and *in vitro* in future studies.

The identification of host cell transcripts differentially expressed in infected and control monolayers is significant given the short duration of the infection. What may explain the rapid transcriptional response is the fast rate of parasite asexual multiplication. At 24 h post-infection, some parasites may already be in second generation of merogony [4]. During merogony, when the rate of parasite replication is at its peak, demand for host cell metabolites, which the parasite is unable to synthesize, and the demand for energy is likely to be high, possibly upregulating related biosynthetic pathways in the host. The fact that such biosynthetic pathways do not feature among those most upregulated in infected monolayers (Additional file 4: Table S2) may indicate that the extra metabolite demand from the developing parasite is not sufficient to impact host cell transcription at a detectable level, or that this demand is met by the host cell through increased activity of biosynthetic enzymes, rather than by upregulating transcription. Since not all cells in a monolayer are infected, the DESeq2 analysis is likely to underestimate the extent of differential expression. *Sus scrofa* genes with the highest level of differential expression are up- or downregulated by about 6-fold (Additional file 3: Table S1, column log2(FC)) indicating that in individual cells, differential expression could be 10-fold or higher.

Attempts to flow-sort populations of infected and uninfected to improve the resolution of differential expression analyses have been reported [12]. The drawback of this approach is that additional manipulations required to release and sort infected from uninfected cells are difficult to standardize and minor differences in processing could potentially affect transcription or mRNA turnover. The limitations of the culture systems also precludes us from distinguishing between transcriptional changes induced by multiplying intracellular meronts from secondary changes triggered, for instance, by the disruption of the monolayer. Gene ontology analysis indicates that genes involved in cell proliferation were upregulated in infected monolayers, possibly indicating a secondary effect triggered by release of cells, loss of contact inhibition and cell re-entering the mitotic cycle. Newer RNA-Seq methods making single-cell RNA-Seq possible might be a viable approach to improve the resolution of transcriptome analysis. Combining this approach with emerging cell culture techniques which support the entire life-cycle [40, 41] could provide access to later stages in the life-cycle and eventually generating a complete picture of parasite and host cell gene regulation during the life-cycle.

The upregulation of genes related to glycoproteins observed in this study was also detected in an earlier microarray analysis [12]. On the other hand, differentially expressed functions reported by others, such as structural proteins and markers of stress, were not found to be upregulated in the present study. This difference could be a consequence of our study using a different cell line, differences in the intensity of the infection, a difference in parasite virulence or perhaps to the signal being below the sensitivity of the assay. As discussed above, the different results generated by quantitative RT PCR, microarrays and RNA-Seq are not surprising. Additional research will be required to generate a validated host cell transcriptional profile in response to *C. parvum* infection.

## Conclusions

The analysis of the transcriptome of cell monolayers infected with *C. parvum* and uninfected controls revealed cellular functions differentially regulated in response to the infection. However, stress- and apoptosis-related genes were not impacted. The comparison of the combined host-parasite transcriptome showed that *C. parvum* gene expression is less diverse and is highly enriched for genes encoding ribosomal functions.

## Additional files

Additional file 1: **Figure S1.** *S. scrofa* FPKM values from two replicate cell cultures infected with *C. parvum* (correlation coefficient *R*^*2*^ = 0.968). (TIFF 56 kb)

Additional file 2: **Figure S2.** Micrographs of immunofluorescently labelled infected (left) and control monolayers of IPEC-J2 cells viewed with 400× magnification. (PDF 28 kb)

Additional file 3: **Table S1.** Host cell genes differentially expressed at a FDR < 0.05. (XLSX 92 kb)

Additional file 4: **Table S2.** KEGG pathways significantly enriched in *C. parvum* infected cell monolayers. (XLSX 15 kb)

Additional file 5: **Table S3.** Pathway analysis of genes upregulated in uninfected monolayers. (XLSX 17 kb)

## Abbreviations

ANOSIM: Analysis of Similarity
FC: fold change
FDR: False Discovery Rate
FPKM: fragments per kilobase of transcript per million mapped reads
PCoA: Principal Coordinates Analysis
RT PCR: reverse-transcription polymerase chain reaction

## References

[1] Kotloff KL, Nataro JP, Blackwelder WC, Nasrin D, Farag TH, Panchalingam S (2013). Burden and aetiology of diarrhoeal disease in infants and young children in developing countries (the Global Enteric Multicenter Study, GEMS): a prospective, case-control study. Lancet.

[2] Shikani H, Weiss LM, Cacciò SM, Widmer G (2014). Human cryptosporidiosis: a clinical perspective. *Cryptosporidium*: parasite and disease.

[3] Griffiths JK (1998). Human cryptosporidiosis: epidemiology, transmission, clinical disease, treatment, and diagnosis. Adv Parasitol.

[4] Current WL, Haynes TB (1984). Complete development of *Cryptosporidium* in cell culture. Science.

[5] Woodmansee DB, Pohlenz JF (1983). Development of *Cryptosporidium* sp. in a human rectal tumor cell line. Proceedings of the Fourth International Symposium on Neonatal Diarrhea.

[6] Striepen B, White MW, Li C, Guerini MN, Malik SB, Logsdon JM (2002). Genetic complementation in apicomplexan parasites. Proc Natl Acad Sci USA.

[7] Deng M, Lancto CA, Abrahamsen MS (2004). *Cryptosporidium parvum* regulation of human epithelial cell gene expression. Int J Parasitol.

[8] Castellanos-Gonzalez A, Yancey LS, Wang HC, Pantenburg B, Liscum KR, Lewis DE (2008). *Cryptosporidium* infection of human intestinal epithelial cells increases expression of osteoprotegerin: a novel mechanism for evasion of host defenses. J Infect Dis.

[9] Yang Y-L, Serrano MG, Sheoran AS, Manque PA, Buck GA, Widmer G (2009). Over-expression and localization of a host protein on the membrane of *Cryptosporidium parvum* infected epithelial cells. Mol Biochem Parasitol.

[10] Zhou R, Hu G, Liu J, Gong A-Y, Drescher KM, Chen X-M (2009). NF-kappaB p65-dependent transactivation of miRNA genes following *Cryptosporidium parvum* infection stimulates epithelial cell immune responses. PLoS Pathog.

[11] Mauzy MJ, Enomoto S, Lancto CA, Abrahamsen MS, Rutherford MS (2012). The *Cryptosporidium parvum* transcriptome during in vitro development. PLoS One.

[12] Yang YL, Buck GA, Widmer G (2010). Cell sorting-assisted microarray profiling of host cell response to *Cryptosporidium parvum* infection. Infect Immun.

[13] Griffiths JK, Moore R, Dooley S, Keusch GT, Tzipori S (1994). *Cryptosporidium parvum* infection of Caco-2 cell monolayers induces an apical monolayer defect, selectively increases transmonolayer permeability, and causes epithelial cell death. Infect Immun..

[14] Widmer G, Corey EA, Stein B, Griffiths JK, Tzipori S (2000). Host cell apoptosis impairs *Cryptosporidium parvum* development in vitro. J Parasitol.

[15] Yu JR, Choi SD (2000). The effect of microfilament inhibitor on the *Cryptosporidium* infection in vitro. Korean J Parasitol.

[16] Yang YL, Serrano MG, Sheoran AS, Manque PA, Buck GA, Widmer G (2009). Over-expression and localization of a host protein on the membrane of *Cryptosporidium parvum* infected epithelial cells. Mol Biochem Parasitol.

[17] Widmer G, Feng X, Tanriverdi S (2004). Genotyping of *Cryptosporidium parvum* with microsatellite markers. Methods Mol Biol.

[18] Widmer G, Lee Y, Hunt P, Martinelli A, Tolkoff M, Bodi K (2012). Comparative genome analysis of two *Cryptosporidium parvum* isolates with different host range. Infect Genet Evol.

[19] Brosnahan AJ, Brown DR (2012). Porcine IPEC-J2 intestinal epithelial cells in microbiological investigations. Vet Microbiol.

[20] Abrahamsen MS, Templeton TJ, Enomoto S, Abrahante JE, Zhu G, Lancto CA (2004). Complete genome sequence of the apicomplexan, *Cryptosporidium parvum*. Science.

[21] Heiges M, Wang H, Robinson E, Aurrecoechea C, Gao X, Kaluskar N (2006). CryptoDB: a *Cryptosporidium* bioinformatics resource update. Nucleic Acids Res.

[22] Schloss PD, Westcott SL, Ryabin T, Hall JR, Hartmann M, Hollister EB (2009). Introducing mothur: open-source, platform-independent, community-supported software for describing and comparing microbial communities. Appl Environ Microbiol.

[23] Kim D, Langmead B, Salzberg SL (2015). HISAT: a fast spliced aligner with low memory requirements. Nat Methods.

[24] Afgan E, Baker D, van den Beek M, Blankenberg D, Bouvier D, Cech M (2016). The Galaxy platform for accessible, reproducible and collaborative biomedical analyses: 2016 update. Nucleic Acids Res.

[25] Trapnell C, Williams BA, Pertea G, Mortazavi A, Kwan G, van Baren MJ (2010). Transcript assembly and quantification by RNA-Seq reveals unannotated transcripts and isoform switching during cell differentiation. Nat Biotechnol.

[26] Love MI, Huber W, Anders S (2014). Moderated estimation of fold change and dispersion for RNA-seq data with DESeq2. Genome Biol.

[27] Peakall R, Smouse PE (2012). GenAlEx 6.5: genetic analysis in Excel. Population genetic software for teaching and research-an update. Bioinformatics.

[28] Clarke KR (1993). Nonparametric multivariate analyses of changes in community structure. Aust J Ecol.

[29] Jiao X, Sherman BT, Huang d W, Stephens R, Baseler MW, Lane HC (2012). DAVID-WS: a stateful web service to facilitate gene/protein list analysis. Bioinformatics.

[30] Benjamini Y, Hochberg Y. Controlling the false discovery rate: a practical and powerful approach to multiple testing. J R Stat Soc Ser B. 1995:289–300.

[31] Hamming RW (1950). Error detecting and error correcting codes. Bell Syst Tech J.

[32] Segata N, Izard J, Waldron L, Gevers D, Miropolsky L, Garrett WS (2011). Metagenomic biomarker discovery and explanation. Genome Biol.

[33] Chen X-M, Gores GJ, Paya CV, LaRusso NF (1999). *Cryptosporidium parvum* induces apoptosis in biliary epithelia by a Fas/Fas ligand-dependent mechanism. Am J Physiol.

[34] Ojcius DM, Perfettini J-L, Bonnin A, Laurent F (1999). Caspase-dependent apoptosis during infection with *Cryptosporidium parvum*. Microbes Infect.

[35] McCole DF, Eckmann L, Laurent F, Kagnoff MF (2000). Intestinal epithelial cell apoptosis following *Cryptosporidium parvum* infection. Infect Immun.

[36] Lumadue JA, Manabe YC, Moore RD, Belitsos PC, Sears CL, Clark DP (1998). A clinicopathologic analysis of AIDS-related cryptosporidiosis. Aids.

[37] Johnson AM, Kaushik RS, Rotella NJ, Hardwidge PR (2009). Enterotoxigenic *Escherichia coli* modulates host intestinal cell membrane asymmetry and metabolic activity. Infect Immun.

[38] Tzipori S, Rand W, Griffiths J, Widmer G, Crabb J (1994). Evaluation of an animal model system for cryptosporidiosis: therapeutic efficacy of paromomycin and hyperimmune bovine colostrum-immunoglobulin. Clin Diagn Lab Immunol.

[39] Akiyoshi DE, Dilo J, Pearson C, Chapman S, Tumwine J, Tzipori S (2003). Characterization of *Cryptosporidium* meleagridis of human origin passaged through different host species. Infect Immun.

[40] Morada M, Lee S, Gunther-Cummins L, Weiss LM, Widmer G, Tzipori S (2016). Continuous culture of *Cryptosporidium parvum* using hollow fiber technology. Int J Parasitol.

[41] DeCicco RePass MA, Chen Y, Lin Y, Zhou W, Kaplan DL, Ward HD (2017). Novel bioengineered three-dimensional human intestinal model for long-term infection of *Cryptosporidium parvum*. Infect Immun.

