# Supplementary figures and images for: Transcriptome analysis of pig intestinal cell monolayers infected with Cryptosporidium parvum asexual stages
Source: Parasit Vectors. 2018 Mar 12;11:176. doi: 10.1186/s13071-018-2754-3 (PMC5848449; doi:10.1186/s13071-018-2754-3)

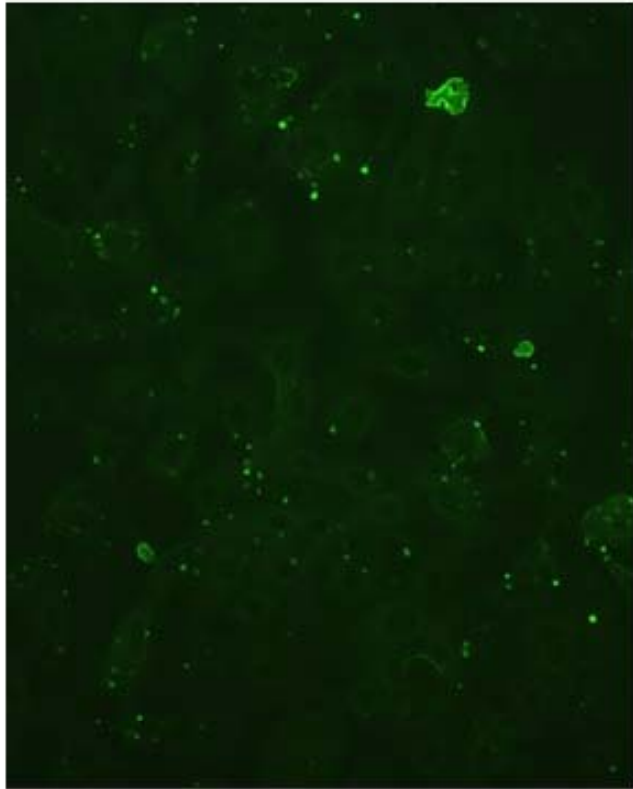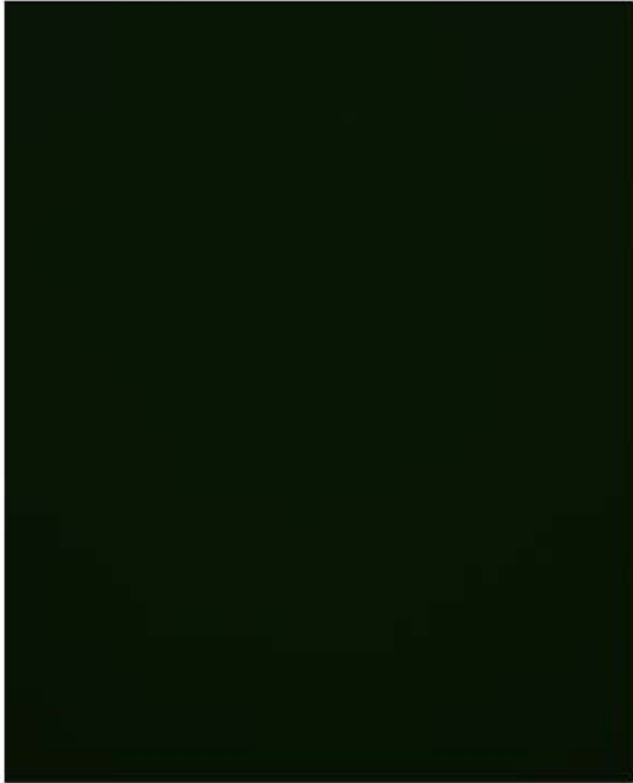

Supplement: Supplementary file 2 — Figure S2. Micrographs of immunofluorescently labelled infected (left) and control monolayers of IPEC-J2 cells viewed with 400× magnification. (PDF 28 kb) [file 13071_2018_2754_MOESM2_ESM.pdf]
